# The Global Dimension of Tomato Yellow Leaf Curl Disease: Current Status and Breeding Perspectives

**DOI:** 10.3390/microorganisms9040740

**Published:** 2021-04-01

**Authors:** Zhe Yan, Anne-Marie A. Wolters, Jesús Navas-Castillo, Yuling Bai

**Affiliations:** 1 Plant Breeding, Wageningen University & Research, P.O. Box 386, 6700 AJ Wageningen, The Netherlands; zhe.yan@wur.nl (Z.Y.); anne-marie.wolters@wur.nl (A.-M.A.W.); 2 Instituto de Hortofruticultura Subtropical y Mediterránea “La Mayora”, Consejo Superior de Investigaciones Científicas Universidad de Málaga (IHSM-CSIC-UMA), Avenida Dr. Weinberg s/n, 29750 Algarrobo-Costa, Málaga, Spain; jnavas@eelm.csic.es

**Keywords:** TYLCD, TYLCV, begomovirus, tomato, *Solanum lycopersicum*, disease resistance, plant breeding

## Abstract

Tomato yellow leaf curl disease (TYLCD) caused by tomato yellow leaf curl virus (TYLCV) and a group of related begomoviruses is an important disease which in recent years has caused serious economic problems in tomato (*Solanum lycopersicum*) production worldwide. Spreading of the vectors, whiteflies of the *Bemisia tabaci* complex, has been responsible for many TYLCD outbreaks. In this review, we summarize the current knowledge of TYLCV and TYLV-like begomoviruses and the driving forces of the increasing global significance through rapid evolution of begomovirus variants, mixed infection in the field, association with betasatellites and host range expansion. Breeding for host plant resistance is considered as one of the most promising and sustainable methods in controlling TYLCD. Resistance to TYLCD was found in several wild relatives of tomato from which six TYLCV resistance genes (*Ty-1* to *Ty-6*) have been identified. Currently, *Ty-1* and *Ty-3* are the primary resistance genes widely used in tomato breeding programs. *Ty-2* is also exploited commercially either alone or in combination with other *Ty*-genes (i.e., *Ty-1*, *Ty-3* or *ty-5*). Additionally, screening of a large collection of wild tomato species has resulted in the identification of novel TYLCD resistance sources. In this review, we focus on genetic resources used to date in breeding for TYLCVD resistance. For future breeding strategies, we discuss several leads in order to make full use of the naturally occurring and engineered resistance to mount a broad-spectrum and sustainable begomovirus resistance.

## 1. Tomato Yellow Leaf Curl Disease Causing Agents: Tomato Yellow Leaf Curl Virus (TYLCV) and TYLCV-Like Viruses

**Figure 1:**
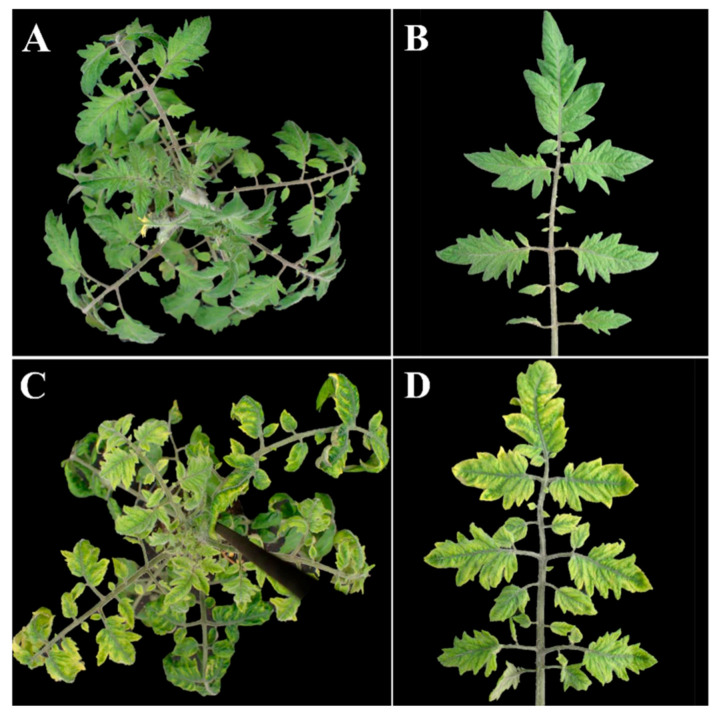
Typical symptoms associated with tomato yellow leaf curl disease. (**A**,**B**) Uninfected tomato plant of cv. Moneymaker. (**C**,**D**) Tomato plant infected with tomato yellow leaf curl virus (TYLCV). Photos were taken 37 days post inoculation using *Agrobacterium*-mediated inoculation of the infectious clone of TYLCV-Israel strain (TYLCV-IL).

A large number of viruses can infect tomato (*Solanum lycopersicum* L.) [1]. These viruses directly or indirectly cause severe reductions in yield and fruit quality. Among them, tomato yellow leaf curl virus (TYLCV) threatens tomato production and currently ranks third after tobacco mosaic virus and tomato spotted wilt virus on the list of the most important plant viruses worldwide [2,3]. TYLCV and 12 TYLCV-like viruses belong to a complex of viruses causing tomato yellow leaf curl disease (TYLCD) [4]. The typical symptoms associated with TYLCD in tomato are leaf yellowing, curling and a marked stunting of plants (Figure 1). At the final stage of disease development, flowers and fruits are abscised followed by cessation of plant growth [5].

**Figure 2:**
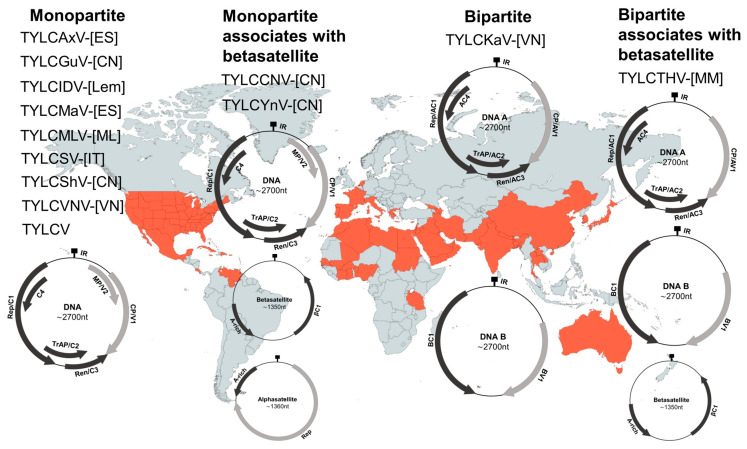
Thirteen virus species causing tomato yellow leaf curl disease (TYLCD) according to the International Committee on Taxonomy of Viruses list of 2019. These viruses are grouped into monopartite and bipartite viruses based on the number of DNA genome components. Their association with alphasatellites and betasatellites are shown. Countries or territories where TYLCV has been officially reported are highlighted in orange-red in the background world map [19]. The complete names of the viruses are tomato yellow leaf curl (TYLC) Axarquia virus (TYLCAxV-[ES]), TYLC-Guangdong virus (TYLCGuV-[CN]), TYLC-Indonesia virus (TYLCIDV-[Lem]), TYLC-Malaga virus (TYLCMaV-[ES]), TYLC-Mali virus (TYLCMLV-[ML]), TYLC-Sardinia virus (TYLCSV-[IT]), TYLC-Shuangbai virus (TYLCShV-[CN]), TYLC-Vietnam virus (TYLCVNV-[VN]), TYLC-China virus (TYLCCNV-[CN]), TYLC-Yunnan virus (TYLCYnV-[CN]), TYLC-Kanchanaburi virus (TYLCKaV-[VN]), TYLC-Thailand virus (TYLCTHV-[MM]).

TYLCD-causing viruses belong to the genus *Begomovirus* in the family *Geminiviridae* [6]. Begomoviruses possess one or two circular single-stranded DNA (ssDNA) genome(s) each of about 2.7–2.8 kb. TYLCV and most TYLCV-like begomoviruses have monopartite genomes consisting of one ssDNA molecule, except for tomato yellow leaf curl Kanchanaburi virus (TYLCKaV) and tomato yellow leaf curl Thailand virus (TYLCTHV). These two begomoviruses are bipartite, with a genome containing two ssDNA molecules, DNA-A and DNA-B (Figure 2) [7,8]. The monopartite TYLCV genome, equivalent to DNA-A of bipartite begomoviruses, contains six open reading frames (ORFs) organized in two transcriptional directions separated by an intergenic region (IR) (Figure 2) [8]. Based on the function, the proteins encoded by the six ORFs have been named: coat protein (CP/V1), virus movement protein (MP/V2), replication-associated protein (Rep/C1), transcriptional activation protein (TrAP/C2), replication enhancer protein (REn/C3) and a protein determining symptom expression and virus spreading (C4) [9]. Bipartite begomoviruses encode the nuclear shuttle protein (BV1/NSP) and movement protein (BC1/MP) on the DNA-B component [10]. All six proteins of monopartite begomoviruses/DNA-A of bipartite begomoviruses and both proteins encoded by the DNA-B component of bipartite begomoviruses are essential for successful systemic infection of host plants [9,10].

Many monopartite begomoviruses including two TYLCV-like viruses, tomato yellow leaf curl China virus (TYLCCNV) and tomato yellow leaf curl Yunnan virus (TYLCYnV), have been shown to associate with satellite DNA molecules, known as alphasatellites and betasatellites (Figure 2) [11,12,13,14]. They are small circular ssDNA molecules of approximately 1350 nucleotides in length. Betasatellites code for one single protein βC1, therefore they rely on helper viruses for replication, cell-to-cell and systemic movement, encapsidation, and insect vector transmission [14]. Emerging evidence shows that co-infection with betasatellites is essential for symptom induction by many monopartite begomoviruses such as TYLCCNV and TYLCYnV [14,15] and enhancing disease severity by a few bipartite begomoviruses, as is the case for TYLCTHV [12,16,17]. Alphasatellites are mainly present associated with monopartite begomoviruses, and are also frequently associated with betasatellites, although their role in infection is not yet fully understood [14,18].

## 2. Global Spreading of TYLCD: Efficient Transmission of Whitefly Vector and Dynamic Nature of the Virus

TYLCD was first reported in the Jordan Valley, Israel, in the late 1930s, and it was not until the 1960s that TYLCV was officially identified as the causal virus of this disease [20]. Since then, the emergence of TYLCD and its subsequent spreading have been extremely rapid into the Mediterranean basin and most tropical and sub-tropical regions of the world (Figure 2) [21,22]. Nowadays, the disease is still spreading to new areas, with recent reported outbreaks in Costa Rica [23] and Trinidad and Tobago [24].

Although TYLCD can be found worldwide, only two strains, the Israel (TYLCV-IL) and Mild strains of TYLCV (TYLCV-Mld), are truly global TYLCD-causing agents [25]. Other begomoviruses associated with TYLCD have been found only in restricted regions, such as TYLCCNV and tomato yellow leaf curl Sardinia virus (TYLCSV) which have been limited to China and Mediterranean countries, respectively [25,26]. The global distribution of TYLCD is closely related to international trafficking/trading of planting material [27], and most importantly to a worldwide increase of the insect vector population and rapid evolution of virus variants [20,25].

### 2.1. An Efficient Transmission Vector: Whiteflies

Under natural conditions, TYLCV is transmitted exclusively by whiteflies (*Bemisia tabaci* Genn.) in either a persistent-circulative [8] or persistent-propagative manner [28]. A single whitefly is able to transmit TYLCV following an acquisition access period of 24 h. In order to reach up to 100% transmission efficiency, 5–15 whiteflies per tomato plant are needed [29,30].

*B. tabaci* is in fact a complex consisting of at least 24 cryptic species that differ in host range, virus-transmitting capacity, host plant adaptation, ability to induce physiological changes, and capacity of spreading and acquiring insecticide resistance [31]. Two species, Middle East–Asia Minor 1 (MEAM1, formerly known as B biotype) and Mediterranean (MED, formerly known as Q biotype), are considered as the most invasive and damaging species, which are also the predominant species that transmit TYLCV to tomato [31,32]. MED has a higher ability to develop insecticide resistance than other species, while MEAM1 is characterized by high fecundity and a wide host range [29,33,34]. Considering that begomoviruses are exclusively transmitted by *B. tabaci*, a change and/or increase of the vector population is one of the key factors associated with the high TYLCD prevalence [7,25]. Taking China as an example, the first invasion of *B. tabaci* MEAM1 appeared in the mid-1990s and was subsequently replaced by MED in 2003. Within a few years after its introduction, MED has become the predominant species in China, has invaded many areas and has been responsible for TYLCD outbreaks [33].

### 2.2. Driving Forces of Begomoviruses Evolution: Mutation and Recombination

TYLCV has a great potential to change due to factors including mutation and genetic recombination which enable rapid adaptation of the TYLCV complex to everchanging environmental conditions [25]. In general, mutation frequency should be lower for ssDNA viruses compared to RNA viruses due to the fact that they take advantage of host DNA polymerases for their replication [27,35]. However, the estimated substitution rates of TYLCV are approximately 10^−4^ to 10^−5^ nucleotide substitutions per site per year which is equivalent to that detected in RNA viruses [27,35]. Although mechanisms triggering high substitution rates found in ssDNA viruses have yet to be fully assessed, it appears that ssDNA viruses are able to escape host DNA polymerase proof-reading repair mechanisms of the replication errors [7,27,35]. By studying the TYLCCNV population [36], it was shown that mutations are not equally distributed along the genome, but are concentrated in the non-coding IR, Rep/C1 and C4 regions. The substitutions of guanine (G) to adenine (A) and cytosine (C) to thymine (T) are dominant for viral populations, whereas the reverse transitions (A to G and T to C) were not detected [36]. Considering that transcriptional gene silencing (TGS) provides a generic response to DNA viruses [37], the methylation directed by the TGS response takes place on cytosine/guanine. Reduced cytosine/guanine levels could, therefore, lead to lower efficiency in methylation.

**Figure 3:**
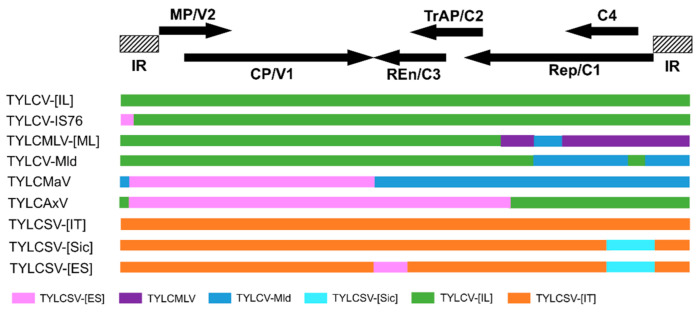
Diagram representing the recombination map of TYLCV complex. Blocks with same color shading represent regions of high identity. The positions of open reading frames (horizontal arrows, CP/V1, MP/V2, Rep/C1, TrAP/C2, Ren/C3, C4) and intergenic regions (IR) are represented on the top of the graph. Tomato yellow leaf curl (TYLC) virus-Israel (TYLCV-[IL]), TYLC Mali virus—Mali (TYLCMLV-[ML]), TYLC virus—Mild [Israel] (TYLCV-Mld [IL]), TYLC Malaga virus-Spain (TYLCMaV-[ES]), TYLC Axarquia virus- Spain (TYLCAxV-[ES]), TYLC Sardinia virus—Italy (TYLCSV-[IT]), TYLC Sardinia virus—Sicily (TYLCSV-[Sic]), TYLC Sardinia virus—Spain (TYLCSV-[ES]).

Recombinant viruses frequently occur in nature. This has contributed greatly to the genetic diversification of TYLCV populations [7,27,38]. Well-documented examples of recombination having an association with (recent) outbreaks and/or epidemics in the Mediterranean Basin and the Middle East are shown in Figure 3. The analysis pinpointed two major groups of the TYLCV complex, one group with TYLCV backbone and the other group with TYLCSV backbone. Several new virus strains have been shown to be recombinants between TYLCV and TYLCSV [7,39,40]. The recombination sites are typically found in the regions of *Rep*/*C1* and *C4* genes, which are referred to as recombination hot spots (Figure 3). Rep/C1 and C4-encoded proteins play an important role in virulence [41,42]. The resulting recombinants are naturally selected for a better fitness through an efficient interaction with host factors [7,27].

## 3. Increasing Global Significance of TYLCD

In the past decades the occurrence of TYLCD has been reported in an increasing number of countries, showing that this major viral disease is still a spreading threat [20,25].

### 3.1. Mixed Infection: An Incubator of New Recombinant Viruses

During mixed infection, a high degree of intra- and inter-species recombination has been observed within the TYLCV complex or among begomoviruses [7]. For example, TYLCV-IL is the result of recombination between TYLCV-Mld and tomato leaf curl Karnataka virus (ToLCKV, a tomato-infecting begomovirus) which occurred in nature during mixed infection [20,43,44]. The Sardinia strain of TYLCSV (TYLCSV-Sar) likely emerged from a South African cassava mosaic virus (SACMV, a cassava begomovirus) ancestor by genetic exchange through recombination [43,45]. Co-infection of tomato plants with TYLCV and TYLCSV led to the emergence of two recombinant viruses associated with TYLCD, tomato yellow leaf curl Málaga virus (TYLCMaV) and tomato yellow leaf curl Axarquia virus (TYLCAxV) (Figure 3), which have acquired a broader host range than either of the parents [25]. A new virus strain (TYLCV-IS76) has arisen due to a recombination event between TYLCV-IL and the Spanish strain of TYLCSV (TYLCSV-ES) (Figure 3) [39,46]. Very recent natural recombinant strains, namely TYLCV-IL [IT:Sic23:16] [47] and TYLCV-IL-[IT:Sar IS141:16] [48], emerged by genetic exchange of parental strains TYLCV and TYLCSV. Both recombinants have been frequently detected in the field in Sicily (Italy) and Sardinia (Italy), respectively [47,48].

### 3.2. Alarming Scenario: Role of Betasatellites in TYLCV Epidemics

Association of TYLCV complex viruses with betasatellites is another factor linked with global TYLCD epidemics. The βC1 protein encoded by betasatellites has been shown to suppress the antiviral RNA interference (RNAi) pathways which provides benefit to the helper viruses for a successful infection [14]. βC1 protein can counteract the post-transcriptional gene silencing (PTGS) pathway by upregulating a Calmodulin-like protein (CaM), which in turn represses the expression of RNA-dependent RNA polymerase 6 (RDR6) by targeting the Suppressor of Gene Silencing 3 (SGS3, a co-factor of RDR6) for degradation [49,50]. βC1 is also able to mediate TGS suppression by physically interacting with and inhibiting S-adenosyl homocysteine hydrolase (SAHH) which is needed to maintain the methylation cycle [51]. In addition, begomovirus-betasatellite co-infection manipulates host insect defense to enhance whitefly behavior and performance. βC1 directly interacts with the transcription factor MYC2 to suppress plant terpene biosynthesis, thereby reducing whitefly resistance [52]. Compared to plants infected with TYLCCNV, plants co-infected with a betasatellite were shown to attract more whiteflies, with female whiteflies laying more eggs, which developed faster into adult whiteflies [52].

Further, betasatellites are capable of being trans-replicated by a wide range of helper begomoviruses in mixed infection. For example, monopartite begomoviruses including the Oman strain of TYLCV (TYLCV-Om) [53], tomato yellow leaf curl Mali virus (TYLCMLV) [54], and TYLCV-IL [55] can trans-replicate betasatellites associated with tomato leaf curl virus (ToLCV), cotton leaf curl Gezira virus (CLCuGV), and honeysuckle yellow vein mosaic virus (HYVMV), respectively. These observations pose an alarming threat that upon polyphagous feeding of whitefly, monopartite begomoviruses may form novel disease complexes by acquiring betasatellites from other begomoviruses [14].

**Figure 4:**
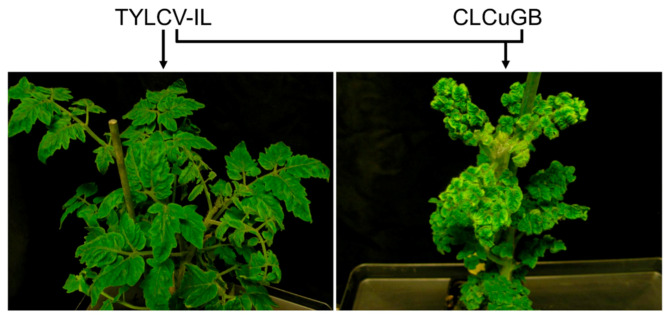
New tomato disease complex with more severe symptoms caused by re-assortments of TYLCV-IL and a betasatellite. Leaf symptoms on tomato plants infected with TYLCV-IL alone or co-infection with CLCuGB (adapted from Conflon et al. [58] with permission). TYLCV-IL: tomato yellow leaf curl virus-Israel; CLCuGB: cotton leaf curl Gezira betasatellite.

Usually the resulting new disease complexes are characterized by more severe symptoms consisting of extremely stunted and distorted plants (Figure 4) [33,55]. In *N. benthamiana*, co-replication of TYLCV with ageratum yellow vein betasatellite (AYVB) increases the symptom severity level [56,57]. Co-infection of TYLCMLV and cotton leaf curl Gezira betallatellite (CLCuGB) by cross-feeding of whiteflies resulted in more severe symptoms [33,54]. In the presence of honeysuckle yellow vein mosaic betasatellite (HYVMVB), TYLCV-infected tomato plants developed more severe stunting symptoms [55]. A recent study showed that co-replication of TYLCV-IL with CLCuGB leads to a significant increase of TYLCV symptoms (Figure 4) [58].

In the Mediterranean basin and the Middle East, which are proposed to be centers of both TYLCV complex origin and diversification, the first betasatellite (i.e., CLCuGB) associated with either TYLCV-IL or TYLCV-Mld in tomato plants was identified very recently in Israel [12,21,59]. This is of great concern for tomato growers worldwide but especially in the Mediterranean region. So far, 61 betasatellite species have been officially reported [4]. China and the Indian subcontinent host more than 90% of betasatellite species. Preventing further spreading of betasatellites to the genetic pool of TYLCV complex can efficiently limit the appearance of new begomovirus-betasatellite disease complexes.

### 3.3. Emerging Problem: Plant Host Range Expansion

TYLCD infection has been detected in 49 plant species including economically important crops and weed species belonging to 16 families [60]. Alternate hosts that act as virus inoculum sources enable the persistence and spread of the virus especially in crop-free periods. Although TYLCV has a diverse host range in addition to tomato, its detection in alternate hosts is rare [61], and TYLCD global spread is associated primarily with tomato [62].

Another important and wide spreading leaf curl disease of tomato is tomato leaf curl disease (ToLCD) [63]. Fifty-five distinct viruses have been associated with ToLCD [4], with one of the most important being tomato leaf curl New Delhi virus (ToLCNDV) which was initially identified in the India sub-continent. ToLCNDV is a bipartite begomovirus species. It causes the most predominant disease affecting tomato and its epidemics were limited to Asian countries [64]. However, in recent years, ToLCNDV has been extending its host range to a broader spectrum, including Cucurbitaceae, Euphorbiaceae, Fabaceae, as well as Malvaceae, and is spreading rapidly to new geographical regions, including the Middle East and the Mediterranean basin [64]. Recent outbreaks of ToLCNDV in the Mediterranean basin have been associated with the emergence of a novel strain, ToLCNDV-ES, which affects mainly cucurbits including cucumber (*Cucumis sativus* L.), melon (*C. melo* L.) and zucchini (*Cucurbita pepo* L.). The isolates of the ToLCNDV-ES strain are well adapted to infect cucurbits, but have limited ability to infect tomato [65,66].

In different begomoviruses, betasatellites also affect the host ranges. For example, cassava, but not ageratum, is the host of the bipartite begomovirus Sri Lankan cassava mosaic virus (SLCMV). However, in the presence of a betasatellite associated with AYVV, SLCMV is also able to infect ageratum and induces typical yellow vein symptoms [14,67].

## 4. TYLCD Control: Mapped TYLCV Resistance Genes

In practice, preventing viruses from infecting the host mainly requires the control of virus vectors by the use of appropriate physical barriers (traps and screens) and chemical agents (insecticides). However, building physical barriers is not always feasible and the application of chemical compounds can result in the development of resistance against the used compound by whiteflies [68,69]. The best crop protection method is host resistance against viruses and/or whiteflies. In tomato breeding for TYLCV resistance, the most prominent approach is transferring virus resistance genes from wild tomato relatives into cultivated tomato.

So far, six resistance genes (*Ty-1*, *Ty-2*, *Ty-3*, *Ty-4*, *ty-5* and *Ty-6*) were identified from a few tomato wild species, including *S. habrochaites* and *S. chilense* (Table 1) [70,71,72,73,74,75]. Four of these TYLCV resistance genes (*Ty-1*/*Ty-3*, *Ty-2* and *ty-5*) have been cloned, representing three classes of antiviral defense mechanisms (Table 1) [76,77,78,79].

*Ty-1* and *Ty-3* originate from *S. chilense* accession LA1969 and LA2779, respectively, and are located on the long arm of tomato chromosome 6 [73,75,80]. They are allelic to each other and encode an RNA-dependent RNA polymerase belonging to the RDRγ type with homology to *Arabidopsis* RDR3, -4 and -5 (Table 1) [78]. *Ty-1* confers resistance to TYLCV based on enhanced TGS by increasing cytosine methylation of the viral genome [81]. *Ty-1*-mediated antiviral TGS response has been considered to be generic against geminiviruses as *Ty-1* was shown to also confer resistance to a bipartite begomovirus tomato severe rugose virus (ToSRV) [81] and the leafhopper-transmitted beet curly top virus (BCTV), a curtovirus [57]. However, co-inoculation with betasatellites expressing the βC1 protein compromises *Ty-1*-mediated resistance and induces disease symptoms [57]. Furthermore, mixed infection with the RNA virus cucumber mosaic virus (CMV) compromises the effectivity of resistance conferred by *Ty-1*, by interference of the CMV RNAi suppressor protein 2b on Argonaut 4 (AGO4) activity involved in TGS [81,82]. The emergence of a recombinant strain TYLCV-IS76 coincides with the increased use of *Ty-1* containing tomato varieties by farmers in Souss (Morocco) [39,46]. TYLCV-IS76 can accumulate better than its parental strains in *Ty-1*-carrying varieties that leads eventually to the displacement of both parental virus strains locally [39,46]. In Sicily (Italy), infection by recombinant variant TYLCV-IL-[IT:Sic23:16] resulted in TYLCD symptoms in tomato plants carrying the *Ty-1* gene. In the same geographical region, samples collected from plants without the *Ty-1* gene harbored a mixture of the recombinant TYLCV-IL-[IT:Sic23:16], and both parental begomoviruses [47]. In contrast, tomato plants with the *Ty-1* gene contain only recombinant genomes. In Sardinia (Italy), recombinant variant TYLCV-IL-[IT:Sar IS141:16] reduces the effectiveness of *Ty-1* mediated resistance as typical TYLCD symptoms were observed on *Ty-1* harboring tomato plants [48]. This variant was found to be positively selected in *Ty-1* resistant plants under field conditions [83]. Collectively, these examples show the limitation of *Ty-1*-mediated resistance. In many breeding programs worldwide, introgression of *Ty-1*/*Ty-3* into cultivated tomatoes has been predominant. Agricultural practices including monocultures (i.e., intensive utilization of *Ty-1*-carrying lines in the Mediterranean basin) are potentially harmful because they may facilitate the emergence of new begomoviruses/disease complexes and the spreading of epidemics [39,46].

*Ty-2* was first reported in a tomato line H24 derived from *S. habrochaites* accession B6013 and was mapped on the long arm of chromosome 11 (Table 1) [71,84]. Fine-mapping of the *Ty-2* gene was a great challenge due to a chromosomal inversion present in *S. habrochaites* compared with *S. lycopersicum*, resulting in suppression of chromosome recombination [85]. By using intraspecific crosses between two *S. habrochaites* accessions, suppression of recombination in the *Ty-2* region was overcome, allowing the fine-mapping of the *Ty-2* gene [85]. *Ty-2* was shown to encode a nucleotide-binding leucine-rich repeat protein (NLR) [77,79]. The Rep/C1 protein of TYLCV presents the Avr determinant of *Ty-2*-based resistance [77].

The *ty-5* gene, a loss-of-function allele of the *Pelota* (*Pelo*) gene encoding a messenger RNA surveillance factor, hinders TYLCV multiplication, leading to resistance in tomato [76]. A tomato inbred line harboring *ty-5* displays resistance to monopartite begomoviruses associated with TYLCD [86] and the bipartite begomovirus tomato chlorotic mottle virus (ToCMoV) [87]. Pelo is involved in the ribosome recycling phase of protein synthesis [76], which is highly conserved among animals, plants and yeast [88]. pelo deficiency in *Drosophila* restricts replication of the RNA viruses cricket paralysis virus (CrPV), Drosophila C virus (DCV) and Drosophila X virus (DXV), and of the DNA virus invertebrate iridescent virus 6 (IIV6) [88]. The *Pelo* gene has also been shown in rice to be involved in resistance against bacterial blight disease caused by *Xanthomonas oryzae* pv. *oryzae* by elevating the salicylic acid pathway [89,90].

In addition to the previously mentioned *Ty*-genes, two other TYLCV resistance genes have been mapped, namely *Ty-4* and *Ty-6*. *Ty-4* originates from *S. chilense* accession LA1932 and has been mapped to the long arm of chromosome 3. *Ty-4* is reported to have a minor effect on TYLCV resistance, accounting only for 15.7% of the total variance [74]. *Ty-6,* originating from *S. chilense* accessions LA1938 or LA2779, is located on the long arm of chromosome 10 [72,91]. *Ty-6* confers moderate resistance to TYLCV, but a high level of resistance to begomovirus tomato mottle virus (ToMoV). The most effective use of *Ty-6* is in combination with other *Ty*-genes such as *Ty-3* or *ty-5* [92].

## 5. Breeding Strategies: Mounting a Broad-Spectrum and Sustainable Begomovirus Resistance

At present, introgression of *Ty-1* or *Ty-3* into cultivated tomato has been the major focus in breeding programs worldwide. However, *Ty-1*-mediated resistance has been observed not to be effective in the field and during mixed infection [39,46,47,48,57,81,93]. Further, the breakdown of *Ty-2*-based resistance was reported by TYLCSV [94] and an isolate of the Mild strain of TYLCV (TYLCV-Mld) [95]. Therefore, efforts have been made to pyramid the *Ty*-genes. At the World Vegetable Centre in Taiwan, tomato lines carrying the *Ty-2* resistance gene in combination with other known *Ty*-genes (i.e., *Ty-1*/*Ty-3* and *ty-5*) have been generated, which are extensively used in breeding programs in many Asian countries and other regions of the world [96,97]. Additionally, there is an urgent need to further exploit wild tomato relatives for novel genes against TYLCD.

### 5.1. Fishing in the Gene Pool: Natural Variation of Wild Tomato Relatives

Germplasm screening for resistance to TYLCD has been performed by researchers worldwide ever since the mid-1980s, when TYLCD became a constraint of tomato production [22,98,99]. The emergence of resistance-breaking recombinant variants like TYLCV-IS76, TYLCV-IL-[IT:Sic23:16] and TYLCV-IL-[IT:Sar IS141:16] are very recent events (appearance in less than 5 years) [39,46,47,48]. As a result, breeders have to search constantly in the genetic pool for effective sources to tackle the rapid evolution of TYLCD-causing agents. The germplasm screened so far is rather extensive, representing a full range of genetic diversity of tomato. Highly resistant accessions exhibiting no TYLCD symptoms have been reported in a number of species (Table 2).

It is clear from these studies that the vast majority of accessions from *S. chilense* are resistant and many resistant accessions can also be found in *S. peruvianum* and other species of its complex (i.e., *S. arcanum*, *S. huaylasense* and *S. corneliomulleri*) (Table 2). In different independent screenings, the highest levels of resistance are found in accessions of these two species. Most of these accessions remain symptomless throughout the whole disease test [97,111,123]. Moreover, resistance in many of these accessions is characterized by reduction in viral accumulation [123]. Wild tomato species (*S. cheesmaniae*, *S. habrochaites*, *S. neorickii*, *S. pennelli*, and *S. pimpinellifolium*) belong to the “esculentum” complex which can be crossed with the cultivated tomato [125]. However, these species generally do not confer a high level of TYLCV resistance (Table 2) [97,116,119].

Next to having a list of symptomless accessions, it is of great importance to determine the virus accumulation levels of symptomless genotypes. This will help to clarify whether the symptomless accessions are also virus-free or not. In tomato varieties containing any of the mapped TYLCV resistance loci (*Ty*-loci), viral replication is not completely blocked since virus accumulation is still detected in systemic tissues [77,91,114,126,127,128]. Clearly, the TYLCD epidemic is extremely difficult to control, but utilization of symptomless virus-free tomato varieties, if possible, could minimize the chances of emergence of recombinant viruses during mixed infection.

In order to identify novel resistance loci, it is important to investigate whether the resistance reported in the symptomless accessions in Table 2 are controlled by allelic variants of the known *Ty*-genes. This may be achieved by comparing the mapping positions of the resistance loci using existing (functional) molecular markers and/or to apply virus-induced gene silencing (VIGS) approaches in combination with allele mining [97].

### 5.2. A Challenging Task: Introgression Breeding for TYLCV Resistance

Many pre-breeding populations and breeding lines have been developed focusing on the introgression of resistance derived from accessions of *S. chilense*, *S. peruvianum*, *S. habrochaites* and to a lesser extent, *S. pimpinellifolium*. *S. pimpinellifolium* is a close wild relative of *S. lycopersicum* that is easily crossable with the cultivated tomato [125]. Therefore, it was the first wild species used to develop TYLCV-resistant lines which in many cases resulted in only partially resistant lines [109,129,130]. In other studies, breeding lines were developed from several *S. pimpinellifolium* accessions that show resistance to TYLCV with no viral symptoms, including: Hirsute-INRA and LA 1478 [99]; LA1582 [131]; LA1921 [98]; PI 407543 and PI 407544 [103]; and G1.1554 (CGN15528) [132]. *S. habrochaites* accessions are also easily crossable with the cultivated tomato [125]. Promising TYLCV-resistant lines were developed using accessions LA1777 and LA0386 [121] and EELM388 and EELM-889 [120].

Despite the high incompatibility, accessions of *S. chilense* and *S. peruvianum* have been well utilized as the most resistant sources. Exploitation of TYLCV resistance in *S. peruvianum* began from the incorporation of the resistance from accession PI 126935 [133] and PI 126944 [113,117,134]. Resistant *S. chilense* accessions (LA1932, LA1938, LA1960, LA1969, LA1971, LA2779 and LA3473) have been intensively applied into breeding practices [92,114,135,136,137]. Previous studies indicated that resistance in these accessions is mediated by functional *Ty-1*/*Ty-3* alleles [97,109,138]. Therefore, attempts to explore and identify new resistance genes using highly resistant *S. chilense* accessions only resulted in the identification of additional *Ty-1*/*Ty-3* alleles. The analyzed functional *Ty-1*/*Ty-3* alleles differ in only a few amino acids [97,138]. Whether each allele displays similar or different characteristics (e.g., durability) remains to be investigated.

### 5.3. Pyramiding Resistance Genes: Towards Durable and Broad-Spectrum Resistance

To produce durable and broad-spectrum resistance, an essential approach would be to use multiple genes, a process known as pyramiding/stacking. Desirably, the stacked genes should confer different types of resistance. The chance of viruses overcoming polygenic resistance is substantially reduced compared to monogenic resistance. It requires a virus to accumulate various mutations with a low incidence and probably a fitness cost to adapt to pyramided resistance genes which is not likely to occur [139]. In light of the effectivity of the *Ty-1* gene against a broad spectrum of DNA viruses, pyramiding distinct gene(s) including *Ty-1* offers one possibility to achieve the goal. Such efforts in tomato have been shown to lead to enhanced resistance relative to the level in the presence of single genes [122,140].

### 5.4. Additional Source for Resistance: Dysfunctional Susceptibility Genes

Susceptibility (*S*) genes encode host proteins exploited by the pathogen to facilitate infection and establish a compatible interaction [141]. Utilization of *S* genes in resistance breeding, therefore, implies impairing their function in disease susceptibility [141]. Impaired *S* genes are known to provide broad-spectrum resistance and are effective not only against many if not all strains or races of a given pathogen, but also against multiple pathogens [141,142]. In addition, the presence and function of numerous *S* genes was found to be conserved between plant species, providing the possibility to impair the *S* gene in different plant species [143]. The *ty-5* gene represents a loss of function allele of the *Pelota* gene [76], showing that the *Pelota* gene is a host susceptibility factor of TYLCV. Loss-of-function mutations at the *Pelo* homologous locus have been shown to provide resistance to a broad range of geminiviruses [144]. In *Capsicum annuum*, plants containing the mutated pepper *Pelo* gene confer resistance to monopartite begomovirus pepper leaf curl virus (PLCV) and bipartite begomovirus pepper yellow leaf curl Indonesian virus (PepYLCIV) [144].

Impaired *S* genes confer resistance in a recessively inherited mode. Recessive resistance against viruses is found with a higher frequency compared to resistance against other types of plant pathogens where most of the reported resistance sources, until now, are dominant [145]. Viruses require many host factors to complete their infection cycle [10]. Many of the naturally occurring recessive resistance genes code for the eukaryotic translation initiation factors (eIF) 4E and eIF4G, and their isoforms. These are effective against RNA viruses [146]. Research on recessive resistance to DNA viruses (geminiviruses) is lagging behind. So far, only a few host factors have been identified as naturally occurring recessive resistant alleles. For example, a recessive gene named *tgr-1* derived from a tomato breeding line has been demonstrated to confer a high level of resistance against ToLCV by impairing viral movement [147]. Resistance to bean golden yellow mosaic virus is controlled by a recessive locus *bgm-1* which reduces mosaic and yellowing symptoms of common bean [148].

Identification of recessive genes is not restricted by the naturally occurring traits only. In practice, if the naturally occurring recessive allele is absent in the gene pool, genetic variation can be created by artificial ways. Discovering host genes involved in viral infection processes can be facilitated by using forward (loss-of-susceptibility mutants) and reverse (candidate gene approach) genetic screens. Attempts to search for genes potentially involved in geminiviral infection can be achieved according to the following criteria: (1) host proteins interacting with geminiviral proteins; (2) host genes exclusively or preferentially expressed in phloem tissues, to which virus is restricted; (3) host genes involved in cellular processes required for geminivirus infection. Using a VIGS-based approach, 11 host genes were identified as involved in TYLCSV infection, with plants showing delayed, reduced or completely abolished infection after silencing [149]. Recent genetic editing techniques such as the clustered regularly interspaced palindromic repeats (CRISPR)/CRISPR-associated genes (Cas) system are widely used to study gene functions [150,151]. This highly specific gene editing technique targeting host susceptibility genes offers plant breeders a unique opportunity to achieve durable resistance against TYLCD- associated viruses.

Forward genetic screening to identify *S* genes is based on artificial mutations. Both chemical and physical mutagenesis are used for this purpose. Among them, the use of the chemical mutagen ethyl methanesulfonate (EMS) is one of the most popular methods to induce large numbers of random point mutations across the whole genome [152]. Over the past years, several EMS tomato populations have been developed using different tomato cultivars [153,154,155,156,157]. The EMS-mutagenized populations can be subjected to phenotypical screening for resistance to TYLCD-causing agents. Cultivated tomatoes do not mount TYLCD defense responses. Once a plant shows no or reduced TYLCD symptoms (compared with the target tomato cultivar), this plant can be further characterized. To confirm the recessive nature of inheritance and find out the causal mutation for the altered phenotype, segregating populations can be developed.

### 5.5. Engineering Virus Resistance: Modification of Virus Genes

Up until now, host plants that are immune to TYLCV infection have never been reported. Breeding for TYLCV complex resistance remains challenging due to the emergence of resistance-breaking strains. To overcome these challenges, conventional transgenic approaches such as pathogen-derived resistance (PDR) has been utilized for improved geminiviruses resistance. This approach involves expression of truncated viral proteins [158] or viral sequences in an inverted-repeat format [159], leading to a resistant phenotype. Engineering *N. benthamiana* and tomato resistance to TYLCSV was accomplished by expression of a truncated TYLCSV-Rep/C1 protein. However, the transgenic plants did not protect against one of the closely related virus strains, TYLCSV-ES, a recombinant derived from TYLCSV which shares 93% amino acid sequence identity [158]. Similarly, expression of a truncated *Rep*/*C1* gene from TYLCV-Mld confers resistance in tomato but not to the TYLCV-IL strain [160]. All the examples demonstrate the limitation of PDR which shows strain specificity at least for Rep/C1 based resistance.

Recent research indicates that durable and broad-spectrum resistance can be achieved using the CRISPR/Cas9 system to target viral genes [161,162,163,164]. First, given the recombination ability of the TYLCV complex in the coding region, the CRISPR/Cas9 system targeting the non-coding intergenic region (IR) reduces the chance of non-homologous end-joining repair (NHEJ)-induced viral variants and enables durable virus interference [161,162]. Second, targeting a conserved sequence of the virus genome allows simultaneous interference with various TYLCD associated species/strains [161,162,163]. Single guide RNA (sgRNA) designed to target a conserved sequence (TAATATTAC) in the IR which serves as the origin of virion-strand DNA replication among geminiviruses and betasatellites of begomoviruses could be an effective approach to combat multiple viruses/virus complex with betasatellites under natural conditions, where mixed infection is commonly observed [161,162,165].

## 6. Conclusions and Prospects

Here, we have described how the ever-changing begomoviruses defeat widely adapted resistant varieties of tomato, spread rapidly throughout the world and expand their host ranges. All reported *Ty*-loci have been shown to allow virus replication though at different extents, defining them as symptomless carriers. The monoculture of resistant tomato varieties is potentially harmful considering that virus-carrying plants serve as reservoirs of new virus variants. Furthermore, the ability of betasatellites to indiscriminately recruit begomoviruses during mixed infections indicates that geographic regions not yet affected are at significant risk and efforts need to be made to control the spread of betasatellites. Sustainable tomato breeding programs can be achieved by pyramiding various genes that cover a diverse range of resistance mechanisms. Meanwhile, efforts to search for new resistance sources either in the large genetic diversity of tomato gene pool or through artificial approaches should be continued.

To summarize, some of the future studies aimed to increase success and durability of genetic resistance to TYLCV and related begomoviruses, in a scenario of globalization, climate change and viral disease emergence, should include:

- Screening of additional wild tomato accessions for natural resistance to TYLCV and related begomoviruses.
- Testing TYLCV-resistant tomato genotypes for resistance to TYLCV-betasatellite complexes.
- Testing the performance of tomato lines containing individual TYLCV-resistant loci (*Ty-1* to *Ty-6*) and their combinations for resistance to other globally emerging begomoviruses, e.g., ToLCNDV.
- Combining the individual *Ty*-genes with whitefly-resistance genes in order to study whether such a combination will prolong the effectiveness of the virus resistance genes.
- Identification of the specific interactions between the proteins encoded by TYLCV (wild or mutants) and the proteins encoded by the *Ty*-gene alleles, as was done for the *Ty-2* gene. This will allow us to forecast the effectiveness and durability of the *Ty*-genes in different tomato production areas by monitoring viral variants in the population.
- Screening for natural and/or induced mutations in *S* genes to get durable resistance to begomoviruses.
- Determining the role of mixed viral infections, more prevalent due to emergence of virus diseases and modifications of crops and vector geographical limits due to climate change, in modulating host resistance and durability.

Altogether, these approaches should enable breeders to achieve durable resistance against TYLCD.

## Figures and Tables

**Table 1:** Tomato (*Solanum lycopersicum*) wild relatives as resistance sources for tomato yellow leaf curl disease.

Resistance Gene ^a^	Genetic Source		Chromosome	Inheritance Pattern	Gene Identity ^c^	Reference
	Accession/Line ^b^	Species				
***Ty-1***	LA1969	*S. chilense*	6	Dominant	RDR	[75,78]
***Ty-2***	B6013	*S. habrochaites*	11	Dominant	NLR	[71,77,79]
***Ty-3***	LA2779	*S. chilense*	6	Dominant	RDR	[73,78]
*Ty-4*	LA1932	*S. chilense*	3	Incomplete dominant		[74]
***ty-5***	Tyking	*S. lycopersicum*	4	Recessive	Pelota	[76]
*Ty-6*	LA2779	*S. chilense*	10	Incomplete dominant		[72]

^a^ Bold font indicates cloned genes; ^b^ Tyking, the source of the *ty-5* gene, is an old tomato cultivar; ^c^ RDR = RNA-dependent RNA polymerase; NLR = nucleotide binding leucine-rich repeat protein; Pelota = Message RNA Surveillance Factor Pelota.

**Table 2:** Summary of previously identified symptomless, symptomatic and segregating accessions to tomato yellow leaf curl virus complex in wild tomato species.

*Solanum* spp. ^a^	Number of Accessions ^b^		
	Symptomless	Symptomatic	Segregating
*S. arcanum*	15	5	5
*S. cheesmaniae*/*S. galapagense*	0	9	0
*S. chilense*	54	6	4
*S. chmielewskii*	1	3	0
*S. corneliomulleri*	30	8	9
*S. habrochaites*	13	52	14
*S. huaylasense*	4	0	0
*S. lycopersicoides*	1	13	0
*S. neorickii*	2	5	2
*S. pennellii*	2	42	2
*S. peruvianum*	69	39	20
*S. pimpinellifolium*	9	455	0

^a^ The number of accessions was summarized from the following articles, [97,98,100,101,102,103,104,105,106,107,108,109,110,111,112,113,114,115,116,117,118,119,120,121,122,123,124]. ^b^ For simplification, phenotypic responses were categorized into three groups, symptomless, symptomatic and segregating. Accessions belonging to the symptomatic category may contain a certain level of resistance/tolerance.
